# Ventilation by mask before and after the administration of neuromuscular blockade: a pragmatic non-inferiority trial

**DOI:** 10.1186/s12871-015-0111-z

**Published:** 2015-10-06

**Authors:** Aaron M. Joffe, Ramesh Ramaiah, Eric Donahue, Richard E. Galgon, Stephan R. Thilen, Charles F. Spiekerman, Sanjay M. Bhananker

**Affiliations:** Department of Anesthesiology and Pain Medicine, University of Washington, Harborview Medical Center, 325 Ninth Avenue, Box 359724, Seattle, WA 98104 USA; University of Washington School of Medicine, Seattle, WA USA; Anesthesiology, University of Wisconsin School of Medicine and Public Health, Madison, WI USA; Department of Oral Health Sciences, University of Washington School of Dentistry, Seattle, WA USA

**Keywords:** Facemask ventilation, Neuromuscular blocking drugs, Airway management, Difficult ventilation

## Abstract

**Background:**

Test ventilating prior to administration of neuromuscular blockade (NMB) in order to avoid a cannot intubate-cannot ventilate situation is a classic anesthesia teaching. The primary aim of our study was to show that facemask ventilation (FMV) after NMB was not inferior to FMV prior to NMB with respect to exhaled gas volumes before and after their administration.

**Methods:**

This study was approved by the University of Washington Human Subjects Division (Seattle, Washington, USA). Written informed consent was obtained from all patients. Measurements of tidal volume (Vte) as well as other respiratory parameters during FMV were made for 60 s after induction of anesthesia and again after NMB. Difficult, impossible, inadequate, and dead-space only mask ventilation was graded using published definitions. Difficult intubation was defined as >2 attempts at intubation. The primary outcome was non-inferiority in Vte during both study periods defined as a mean difference of <50 mL. Multivariate analysis was performed to assess for interaction between operator experience, patient risk factors for difficult mask ventilation, exhaled volumes, and use of airway adjuncts.

**Results:**

Two-hundred and ten patients were studied. Overall, FMV improved after NMBD. The mean (SD) V_te_ in mL/breath increased from 399 (169) to 428 (166) (mean dif. 30 mL, *p* = 0.001) and the minute ventilation in L/min from 5.6 (2.5) to 6.3 (2.5) (mean dif. 0.6, *p* < 0.001). No patient who was difficult to ventilate after induction became impossible after NMB.

**Discussion:**

In patients at risk for or judged to be a difficult FMV by clinical grading scales, tidal volumes improved after administration of NMBDs. None of these patients exhibited a decline in ventilation or became impossible to ventilate after NMBDs. Several limitations are noted, including the use of hand-delivered breaths and inability to account for time-related changes in ventilation conditions independent of NMBDs.

**Conclusion:**

We conclude that FMV is no worse after NMB than before and is likely to improve airway conditions.

**Trial Registration:**

ClinicalTrials.gov Identifier: NCT02237443. Registered August 28, 2014

**Electronic supplementary material:**

The online version of this article (doi:10.1186/s12871-015-0111-z) contains supplementary material, which is available to authorized users.

## Background

Facemask ventilation (FMV) is considered the most basic of airway management skills and the first-line technique to provide ventilation of the unconscious and apneic patient. While several patient-related factors have been identified as high-risk features for difficult or impossible mask ventilation (DMV or IMV) after induction of [1–4], whether or not the routine administration of a neuromuscular blocking drug (NMBD) prior to “testing” the operators’ ability to ventilate the patient by mask is a help or a hindrance is debatable [5]. Those who argue for “testing” the ability to ventilate before administering NMBD cite concern that complete airway obstruction from NMBD-induced upper airway collapse could result in a “cannot intubate-cannot ventilate” situation if tracheal intubation were not possible [6–8]. Still, others argue that residual muscle tone after the induction of anaesthesia results in some resistance to mask ventilation that is only interpreted by the operator as DMV [9]. If true, administration of a NMBD just after intravenous induction agents would facilitate ventilation as well as allowing earlier identification of patients who will be difficult or impossible, allowing for more timely intervention to establish an airway. Investigators have reported either no effect [10] or improvements in mask ventilation difficulty scores [11] and exhaled volumes [12, 13] after NMBDs have been administered. Notably, none of these studies have reported deterioration in FMV after the administration of a NMBD. However, these studies were designed to demonstrate the superiority of FMV after NMBD with respect to exhaled volumes and thus, may have been underpowered to demonstrate clinically relevant side effects, chiefly, worsening of FMV associated with hypoxia and/or difficult intubation. Thus, the primary aim of our study was to demonstrate the non-inferiority of NMBDs administered to facilitate mask ventilation with respect to exhaled tidal volumes measured before and after their administration.

## Methods

The trial was registered with ClinicalTrials.gov (identifier NCT02237443) and approved by the University of Washington Human Subjects Division (Seattle, Washington, USA). Written informed consent was obtained from all patients or their legal surrogate. All adults (≥18 years of age) were eligible if all of the following conditions were met; they were scheduled for elective or semi-elective surgery, induction of general anaesthesia by intravenous propofol was planned, a tracheal tube was to be placed for airway maintenance. Patients who had symptomatic reflux, prior oesophagectomy, hiatal hernia, emesis within 24 h of surgery, an oropharyngeal or facial pathology making a proper mask fit unlikely, a prior allergy or contraindication to receiving rocuronium bromide, vecuronium bromide, or succinylcholine chloride, or any condition for which the primary anaesthesia team deemed a rapid-sequence intubation to be appropriate, or who were pregnant were excluded.

Patient enrollment was not consecutive, but rather a convenience sample. A study coordinator and an investigator examined the surgical case list the evening prior to or on the morning of the planned surgery. An investigator then informed the anaesthesia team responsible for potential participants of their patient’s study eligibility. At the discretion of the primary anaesthesia team, permission was obtained for the study coordinator to approach the patient for consent to participate on the day of surgery.

All pre-medications and the doses of intravenous medications given to induce anaesthesia were at the sole discretion of the primary anaesthesia care team. Once inside the operating room (OR), standard American Society of Anesthesiologists (ASA) monitors were established. Patients were placed supine, with their head in a sniffing position using standard pillows. Patients with a body mass index > 30 kg.m^−2^ were positioned in a 10 % or greater head elevated position using the controls of a standard OR table or wedge. Patients were pre-oxygenated by tidal breathing 8–10 liters/minute of 100 % oxygen for a period of 2–3 min. Anaesthesia was induced with intravenous propofol with or without the concomitant administration of intravenous fentanyl. Use of volatile agents was allowed. Study measurements began once the plane of anaesthesia was deep enough to render the patient unresponsive to a vigorous jaw thrust and the anaesthesia provider indicated to the investigator that they were ready. Depth of anaesthesia monitoring was not used.

**Fig. 1:**
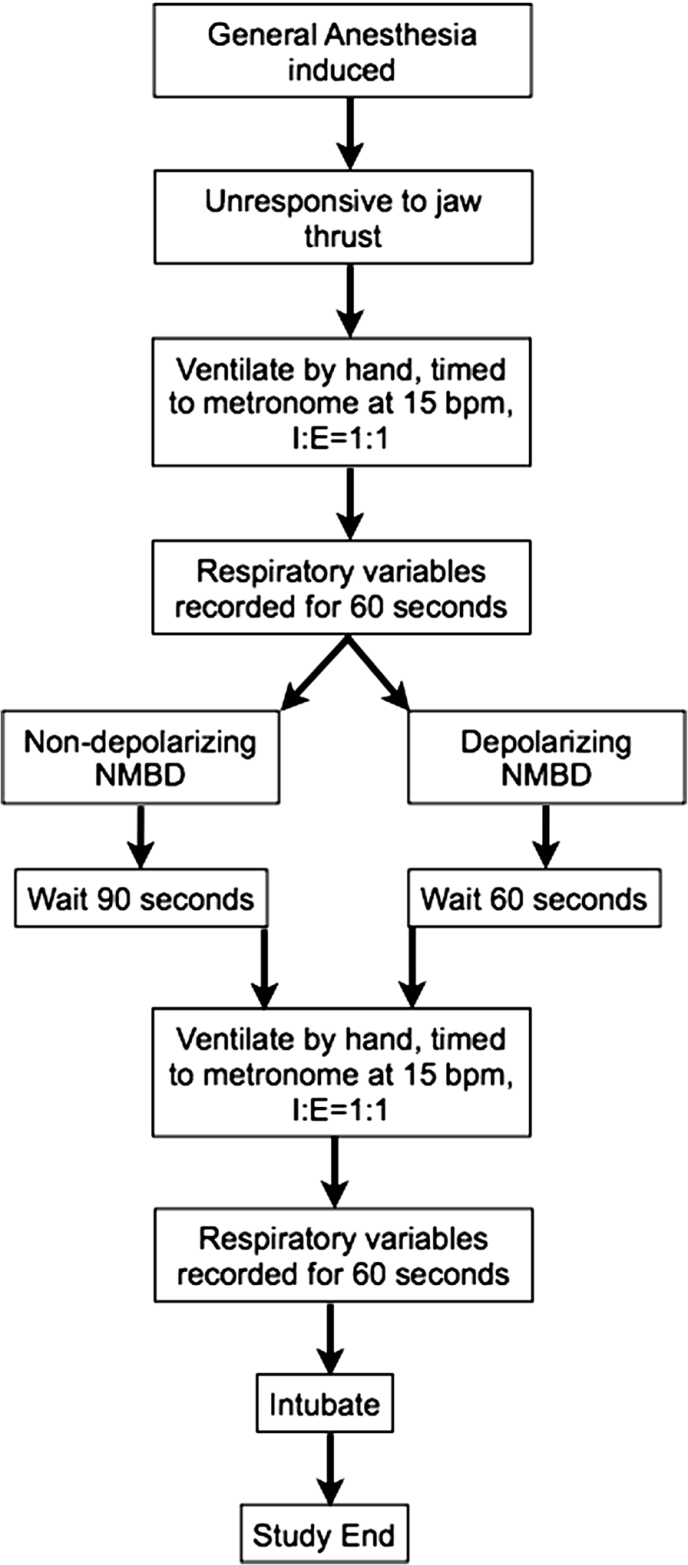
Diagrammatic representation of patient flow through study protocol

Facemask ventilation was performed using a generic single-handed technique, which incorporated a chin-lift head-tilt manoeuvre via an appropriately sized adult facemask (Anaesthesia Face Mask by Vital Signs, Medline Industries, Mundelein, IL). An audible metronome was used to provide a timing prompt in order to achieve a respiratory rate of 15 breaths per minute with an inspiratory-to-expiratory ratio of 1-to-1. Per usual care, providers were instructed to maintain peak airway pressure < 20 cmH_2_O, as displayed by the pressure manometer on the anaesthesia machine. Use of oro- or nasopharyngeal airways, two-hand jaw thrust technique with a second operator squeezing the breathing bag, and repositioning of the airway to maximise upper airway patency during each measurement period was at the discretion of the airway managers; however, an algorithm for dealing with increasing levels of difficulty with facemask ventilation based on typical practice was suggested (see Additional file 1). Exhaled tidal volumes (V_te_) in ml and peak inspiratory airway pressure (PIP) in cmH_2_O were measured using a FloTrak Elite sensor (Phillips Healthcare, Andover, MD) placed in-line with the anaesthesia circuit at the patient wye and attached to a Respironics NM3 respiratory monitor (Phillips Healthcare, Andover, MD) for display and recording purposes. Data was stored real-time to a USB storage device and then imported into Microsoft Excel® for Mac 2011 v14.4.4 (Microsoft Inc., Redmond, WA). At a V_te_ of 40–2500 ml and PIP up to 120 cmH_2_O, the device is accurate to within the greater of 10 ml or 5 % of the reading and the greater of 0.5 cmH_2_O or 2 % of the reading, respectively.^1^ Providers performing the FMV could see the anaesthesia monitor displaying vital signs, including pulse oximetry (SpO_2_), end-tidal carbon dioxide (ETCO_2_), inhalation anaesthetic concentration, and pressure manometer of the anaesthesia machine, but were blinded to inspiratory/expiratory gas flows. Respiratory data was recorded for one minute after induction of general anaesthesia (the “before” NMBD study period). A NMBD was then administered. Recording was performed for another minute after waiting 60 s post succinylcholine administration or 90 s post rocuronium or vecuronium administration, respectively (the “after” NMBD study period). In accordance with local practice, monitoring neuromuscular function was not used during induction of anaesthesia. Patient flow through the study is shown in Fig. 1.

Difficult and impossible mask ventilation (DMV and IMV) was defined on the basis of clinical criteria using the Han’s score [14] and Warters score [11]. Inadequate mask ventilation (MV_i_) was defined as an average returned tidal volume (V_te_) of < 4 ml.kg^−1^ of predicted body weight (PBW) while dead-space ventilation (V_ds_) was defined as an average returned V_te_ of < 150 ml.breath^−1^ associated with clinical signs (inadequate chest rise, no fogging in the mask, no positive tracing of end-tidal carbon dioxide and/or lack of measurable returned tidal volumes on the anaesthesia monitor) [15]. Predicted body weight was calculated using the National Heart, Lung and Blood Institute Acute Respiratory Distress Syndrome Network formula.^2^ Difficult intubation, defined as >2 attempts at intubation using any technique, was also noted. The study could be terminated at the discretion of the primary anaesthesia team if ventilation was inadequate by the above mentioned clinical criteria, if the SpO_2_ was < 90 %, or if the primary anaesthesia team felt for any reason that immediate tracheal intubation was needed.

Baseline patient characteristics, including age, sex, height, weight, and ASA physical status were recorded. Each participant underwent an independent airway exam by the study coordinator in addition to that of the primary anaesthesia team prior to induction. Risk factors for DMV, including Mallampati grade, presence of facial hair, the ability to and extent of voluntary mandibular protrusion, lack of dentition, limited cervical spine motion, or a large or extremely wasted face were noted. In the event of discordance between exams, a study investigator adjudicated findings. The experience/training level of the airway manager was also recorded.

Our primary outcome was non-inferiority in the average V_te_ per breath (over one minute) during both study periods defined as a mean difference of <50 ml. Secondary outcomes were the difference in total minute ventilation (VE) in l/min (calculated as the sum of all breaths delivered during each one-minute study period), the occurrence f DMV (by Han’s or Warters’ scale), and a composite of MV_i_ and/or V_ds_ before and after the administration of NMBDs.

### Statistical analysis

At the time of the planning of this study, no studies had reported a mean (SD) for V_te_ before and after administration of NMBDs. However, based upon a prior comparative trial of two different mask-hold techniques^15^, the SD of the average V_te_ after induction of anaesthesia, using the anaesthesia ventilator and a two-hand jaw-thrust mask hold technique was 130 ml. With the equivalence limit, d, set as 50 ml per breath, with a significance level (α) of 2.5 % and a power (1-β) of 95 %, a total sample size of 208 patients per group (before and after NMBD for a total sample of 416 study periods) were required. The primary outcome (V_te_), as well as other paired continuous data was compared by paired t-test or Wilcoxon signed rank test depending on whether the data were normally distributed. Mean differences are reported for normally distributed continuous data only. Paired categorical data were analysed using McNemar’s test for paired proportions. In order to account for potential confounding of the primary outcome by operator experience, the number of pre-existing patient risk factors for DMV, and use of airway adjuncts (a second operator, oral or nasopharyngeal airways), multiple regression analyses were performed comparing the after to before measurements adjusting for the characteristics listed. Because the use of airway adjuncts could differ between the before and after observations, the unit of analysis for this model was patient observation, in this case two observations (before and after) per patient. The Generalised Estimating Equations approach was used in computing the regression models, which uses a robust variance estimator to account for the correlation between observations from the same patient. The parameter of interest is the before-after NMBD covariate.

Data were analysed using R version 2.9.1 (R Foundation for Statistical Computing, Vienna, Austria). Unless otherwise noted, data are presented as mean (SD), median (IQR [range]) or frequency (%). Statistical significance was defined as a two-sided p-value <0.05.

## Results

**Fig. 2:**
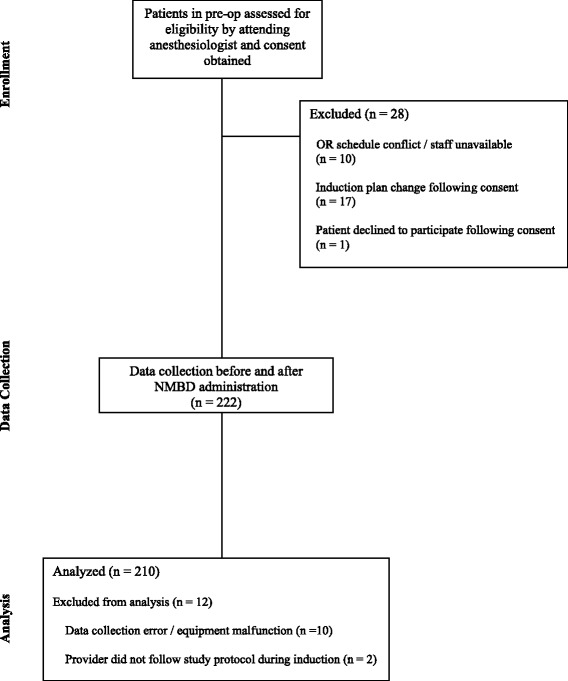
Consort diagram of patient flow through the study

Two hundred and ten patients were studied (see Fig. 2). Baseline characteristics and the prevalence of specific risk factors for DMV in the study population are presented in Tables 1 and 2. Anaesthesia was induced with 2.13 (0.64) mg.kg^−1^ of propofol. One hundred and eighty-six patients also received 1.32 (0.81) mcg.kg^−1^ of fentanyl as part of their anaesthetic induction. Paralysis was induced with 0.6 (0.15) mg.kg^−1^ of rocuronium in 190 patients, 0.75 (0.25) mg.kg^−1^ of succinylcholine in 11 patients, and 0.09 (0.02) mg.kg^−1^ of vecuronium in 9 patients. Fifty-nine individuals provided anaesthesia. Experience level of the airway mangers and how many cases they managed are shown in Table 3.

**Table 1:** Baseline characteristics of 210 patients undergoing face mask ventilation before and after neuromuscular blockade. Data are presented as number (%), mean (SD), or median (IQR [range])

Age, years	45 (17)
Male gender	135 (64.3)
ASA physical status	2 (1–2[1–4])
Height, cm	173 (13)
Weight, kg	85 (22)
BMI, kg.m^−2^	27 (23-32[15–53])
Risk factors for DMV	
None	36 (17.1)
1	68 (32.4)
2	64 (30.5)
3	28 (13.3)
4	12 (5.7)
≥5	2 (1)

*ASA* American Society of Anesthesiologists, *BMI* body mass index, *DMV* difficult mask ventilation

**Table 2:** Prevalence of specific risk factors for difficult mask ventilation among patients undergoing face mask ventilation before and after neuromuscular blockade. Data are presented as number (%)

Age >55 years	70 (33.3)
BMI >30 kg.m^−2^	71 (33.8)
Mallampati grade ≥3	62 (29.5)
Cannot prognath	14 (6.6)
Facial hair	30 (14.2)
History of snoring or OSA	39 (18.6)
Edentulous	13 (6.2)
Limited cervical spine extension	21 (10)
Large or wasted face	23 (11)

*BMI* body mass index, *DMV* difficult mask ventilation, *OSA* obstructive sleep apnea

**Table 3:** Characteristics of anaesthesia who provided face mask ventilation to patients before and after neuromuscular blockade. Data are presened as number (%) or median (IQR [range])

Number of providers	59
Male gender	136 (65)
Finger span, cm	22 (21-23 [17.5-26])
Dominant hand, right	192 (91)
Experience level	
CA-1	39 (18.6)
CA-2	64 (30.4)
CA-3	11 (5.2)
Fellow	8 (3.8)
Attending	10 (4.7)
CRNA	78 (37.6)

*CA* clinical anaesthesia training year, *CRNA* nurse anaesthetists

The results for primary and secondary outcomes are presented in Table 4. With respect to the primary outcome, the average V_te_ in ml/breath before and after the administration of a NMBD was 399 (169) and 428 (166) (mean difference (95 % CI) = 30 (12, 47);*p* = 0.001). Average V_E_ was also greater (5.6 (2.5) vs 6.3 (2.5), mean difference = 0.6 (0.4, 0.9); *p* < 0.001) after NMBDs.

**Table 4:** Comparison of selected outcomes of face mask ventilation before and after administration of neuromuscular blocking drugs. Data presented as mean (SD), median (IQR [range]), or number (%)

	Before	After	Mean difference (95 % CI)	*p*-value^a,b^
Vt, ml.breath^−1^	399 (169)	428 (166)	30 (12, 47)	0.001
PIP, cmH_2_O	19 (5.5)	17 (3.4)	−1.7 (−1, −2.4)	<0.001
Breaths delivered	14 (2.2)	15 (1.5)	---	<0.001
V_E_, l.min^−1^	5.6 (2.5)	6.3 (2.5)	0.6 (0.4, 0.9)	<0.001
Han scale	1 (1-2 [1–4])	1 (1-2 [1–4])	---	0.11
Warters scale	5 (0-6 [0–10])	2 (0-5 [0–9])	−0.7 (−0.4, −1)	<0.001
MV_i_ or V_ds_	35 (16.6)	27 (12.8)	---	0.20

Predicted body weight based on height in inches and gender from National Heart, Lung and Blood Institute Acute Respiratory Distress Syndrome Network formula, which can be accessed at http://www.ardsnet.org/files/pbwtables_2005-02-02.pdf

^a^Comparisons made by paired t-test or Wilcoxon signed rank test for normally and non-normally distributed paired continuous data, respectively. Mean difference displayed for normally distributed continuous data only.^b^Comparison made by McNemar’s test for paired proportions of binomial data

*Vt* tidal volume, *PIP* peak inspiratory pressure, *CI* confidence interval, *SD* standard deviation, V_E_ minute ventilation, MV_i_ inadequate mask ventilation (<4 ml.kg^−1^ predicted body weight), V_ds_ dead-space ventilation (<150 ml, no clinical sign of ventilation)

The proportion of patients in whom an airway adjunct/assist was used (oral or nasopharyngeal airways, a second operator, two-hand jaw thrust technique) was similar before and after NMBDs (28.5 % versus 30.4 %, *p* = 0.34). After adjustment for gender, operator experience, pre-existing patient risk factors for DMV, and use of airway adjuncts (a second operator, oral or nasopharyngeal airways), the results were essentially unchanged. The effect of NMBDs was to increase V_te_ by 31 ml.breath^−1^ (95 % Wald CI =13, 48; *p* = 0.001) and to increase V_E_ by 0.7 l.min^−1^ (95 % Wald CI =0.4, 0.9; *p* < 0.001). No patient suffered oxygen desaturation or <90 % during the study period. No gastric distension was noted by either auscultation or abdominal distension at any time during FMV in any of the study patients.

In post-hoc analysis, degradation of FMV was noted in some patients after the administration of NMBDs. Nineteen percent (40/210) of patients had a decrease in V_te_ outside of the predetermined equivalence limit of 50ml (495 (170) before versus 351 (138) after NMBD, mean difference 82 ml; 95 % CI 57–105, *p* < 0.001). Although these patients were slightly older than those in whom FMV did not degrade after NMBD (46 (17) versus 40 (18) years old, *p* = 0.03), sex of the study participants, ASA physical status, height, weight, BMI, the number of patients with multiple risk factors for DMV as well as the overall proportion of patients with each risk factor, were similar. Further, no patient who was considered difficult by any of the scales used became impossible after the administration of NMBDs. Additionally, among the subgroup of patients who were initially considered as DMV/IMV or MV_i_ /V_ds_, none suffered a degradation in V_te_ and, overall, the V_te_ improved significantly after NMBD administration (Table 5). Three patients (1.4 %) were defined as difficult intubations (requiring >2 attempts at intubation). All 3 patients were intubated by the third attempt and none of them were difficult to hand ventilate before or after administration of the NMBD.

**Table 5:** Comparison of tidal volumes before and after administration of neuromuscular blocking drugs in patients who were initially graded as difficult face mask ventilation

	N	Before	After	Mean difference (95 % CI)	*p*-value^*^
Han score ≥3	32	350 (170)	430 (200)	77 (26, 130)	0.004
Warters score ≥4	119	320 (130)	370 (130)	46 (26, 70)	<0.001
Vt < 4 ml.kg^−1^ PBW or <150 ml	39	170 (74)	290 (120)	110 (69, 150)	<0.001

^*^Comparisons made by paired t-test

V_t_ tidal volume, *PBW* Predicted body weight

## Discussion

Our main findings are that FMV after administration of NMBDs is not inferior to FMV after induction alone. Further, in a pre-defined subgroup of patients in whom FMV was judged to be difficult by a Han’s score ≥3 or Warters’ score ≥4, Vte was significantly improved after administration of NMBDs. Similarly, patients definded as having inadequate ventilation (V_te_ < 4 ml.kg^−1^ PBW) after induction of anaesthesia also showed improved ventilation after the administration of NMBDs. Importantly, none of these patients exhibited a decline in ventilation or became impossible to ventilate after NMBDs. Our findings are consistent with prior investigations [11–13]. However, only 2 of these studies reported actual exhaled gas volumes. Ikeda and colleagues reported an increase in tidal volumes from 4.2 (2.1) to 5.4 (2.6) ml.kg^−1^ (*P* = 0.02) in 17 patients following succinylcholine administration [12]. In a larger and more recent trial, Sachdeva et al. reported that in 125 patients administered rocuronium post-induction, average tidal volumes increased from 525 (116) ml to 586 (129) ml (*p* < 0.001) [13]. In contrast to our findings, neither of these prior investigations noted any degradation in ventilation after the administration of NMBDs. A number of explanations for this discrepancy exist. First, our study was much larger than any similar prior investigation and has greater power to detect low frequency events. Second, having a normal airway or a history of a difficult airway was not needed for inclusion or exclusion in our study, respectively. Indeed, half of our study population had multiple risk factors for DMV. In particular, approximately one-third of our patients had a BMI >30 (10 patients had a BMI >40, range 41–53 kg.m^−2^) and/or a MP grade of >3. Third, in our study patients’ lungs were hand-ventilated using the breathing bag of the anaesthesia machine rather than reciveing breaths mechanically generated via the anaesthesia ventilator. Without airway pressures held constant throughout the respiratory cycle, tidal volumes may have varied from breath to breath. Use of the anaesthesia ventilator to deliver breaths in pressure control mode frees the anaesthesiologist to use two hands for performing airway maneuvers, which has been documented to result in greater ventilation than use of a typical left-handed “EC-clamp” technique [15]. Still, the results of our multivariate analysis, which adjusted for DMV risk factors, operator experience, and use of airway adjuncts showed a similar advantage of NMBDs on FMV, regardless of whether it was the tidal volume or overall minute ventilation was considered.

In order for our study to have greater practical application to bedside clinicians, we graded the ease of FMV before and after administration of NMBDs using currently published definitions in addition to recording exhaled gas volumes. We considered the Han, Warters, and MV_i_/V_ds_ definitions to be clinical [14], clinico-physiologic [11], and physiologic [15]. It is interesting that improvement of FMV was noted after administration of NMBDs with one clinical grading scale (Warters), but not another (Han). The explanation lies in how each of the scales is constructed. The Han scale is ordinal, categorized as grade 1 (easy mask ventilation) to grade 4 (impossible mask ventilation), and is mostly subjective [14]. There is little difference between a grade 2 (needs OPA oral or adjuvant with or without NMBDs) and a grade 3 (difficult - inadequate or unstable or requiring two care providers, with or without NMBDs). As a result, small, but potentially relevant differences in mask ventilation conditions are difficult to quantify. In contrast, the Warters grading scale is a composite score based on the ability to achieve a target tidal volume of 5 ml.kg^−1^ [11]. Points are awarded based on the need for a NPA, OPA, second operator, increasing PIPs, or tidal volumes < 5 ml.kg^−1^. Our observation that patients, on average, received greater tidal volumes with lower PIPs after administration of NMBDs provides the most plausible explanation for the significant decrease in Warters scores between the two measurement periods.

Irrespective of which definition used, overall, patients who were difficult to ventilate achieved statistically greater tidal volumes following the administration of a NMBD. On average, the increase in exhaled volumes was 46–110 ml. As noted by other investigators [13], this increase should not be discounted solely as one of statistical relevance. Based on a respiratory rate of 15 breaths/min, as we suggested in our study, minute ventilation would increase by 690–1650 ml/min, an amount that is greater than two-times the average oxygen consumption in an otherwise healthy patient [16]. Thus, we conclude that this difference is clinically relevant.

We acknowledge that our study has limitations. We employed convenience sampling, which could have introduced selection bias. The only portion of the study protocol that was mandated was the timing between anaesthetic induction and administration of the NMBD. Positioning of the airway and the use of airway adjuncts to maximise upper airway patency were left to the discretion of the primary anaesthesia providers. Also, as already stated, a standard airway pressure profile throughout the respiratory cycle, as would be the case when using the mechanical ventilator of the anaesthesia machine in pressure control mode, was not provided. Thus, some of the differences observed between study periods could be independent of the effects from the NMBDs. While study methods standardising every aspect of airway managment may have been more scientifically valid, we believe the generalisability to routine anaesthetic practice would have been severely limited. Using mechanically generated breaths from the anaesthesia ventilator rather than the breathing bag is much less common. It should also be noted that the most commonly used FMV grading scale, the Han scale [14], was derived from patients who were hand ventilated. The same is true of the Warter’s scale [11]. Furthermore, the most widely cited studies on the incidence of DMV, IMV, DMV/IMV, or DMV combined with difficult laryngoscopy are reporting on patients who were hand ventilated after the induction of anaesthesia [1–4, 17].

Regarding the use of each patient as there own control, we cannot know what would have happened to the tidal volumes over time without the NMBDs. FMV may improve over time due to deepening in the plane of anaesthesia. In our study anaesthesia was induced intravenously using propofol with or without the concomitant administration of fentanyl. While we did assess depth of anaesthesia clinically prior to initiating FMV, we did not employ specific depth of anaesthesia monitoring. Thus, we cannot exclude the possibility that some of the DMV we observed was a result of a suboptimal depth of anaesthesia. Additionally, uncontrolled adjustments in airway positioning made by the operator to optimise ventilation may have also improved FMV even without administration of NMBDs. This limitation could be overcome by randomising patients to receive a NMBD or placebo after the initial, post-induction measurments are recorded. However, our primary goal was to demonstrate that in any individual patient, the administration of a NMBD would not turn a DMV scenario into one where ventilation is not possible. Thus, we believe our study design, where the use of NMBDs is within the context of routine anaesthetic practice, is justified.

In the operating room, DMV is uncommon (<1.5 per 100 mask ventilations [3]) and IMV is rare (1.5 per 1000 mask ventilations [4]). The combined incidence of DMV and one of the following: inability to intubate by DL, DL with a bougie introducer, or videolaryngoscopy is rare (0.04 % or 1 per 2,500 anaesthetics [17]). Even so, the incidence of DMV or DMV combined with DL has been reported to increase in patients who have not been administered a NMBD [17]. It has been noted that a major limitation of the studies just mentioned is that they are based on large observational datasets extracted from the electronic medical record. Thus, the specific timing and effect of any administered NMBDs cannot be specifically examined. As a result, controlled experiments specifically designed to assess the impact of NMB on DMV are needed [17]. While our study was not large enough to document equivalence for an outcome of “cannot-intubate, cannot ventilate,” it is the largest to date within the context of other similar studies [11–13]. Importantly, although we found that FMV did degrade in some patients, none became impossible to ventilate and all were easily intubated. This adds to the literature, which supports, but does not confirm the safety of giving NMBDs at the outset of anaesthetic induction in patients where tracheal intubation is planned to optimise intubating conditions. Lastly, the reader is cautioned not be generalise our results to patients not represented by our study population. In particular, children, parturients and true difficult intubations were not studied.

## Conclusion

We set out to show that FMV before and after the administration of a NMBD would not, on average, cause a decrease in delivered tidal volumes greater than an *a priori* defined equivalence limit of 50 mL. We found that in our study population of elective surgical patients, half of whom exhibited multiple risk factors for DMV and a quarter of whom had a BMI >32 kg.m^−2^, the administration of NMBD did not make FMV worse. In fact, the administration of a NMBD was more likely than not to facilitate FMV. Furthermore, only 3 patients required a third intubation attempt, none of who were difficult to mask ventilate. Thus, routinely “testing” the operators’ ability to ventilate the patient by mask prior to the administration of a NMBD would appear to provide little diagnostic information. However, anaesthesiologists must still use their best judgment in integrating their individual skills set and the patient’s airway characteristics when deciding on the the best course of action.

## Additional files

Additional file 1:
**Suggested algorithm for dealing with increasing levels of difficulty with facemask ventilation.** (TIFF 3 mb)

## Footnotes

1 http://oem.respironics.com/Downloads/FloTrakElite020513.pdf. Accessed October 1, 2014.

2 http://www.ardsnet.org/files/pbwtables_2005-02-02.pdf. Accessed October 1, 2014.
