# Supplementary figures and images for: Ventilation by mask before and after the administration of neuromuscular blockade: a pragmatic non-inferiority trial
Source: BMC Anesthesiol. 2015 Oct 6;15:134. doi: 10.1186/s12871-015-0111-z (PMC4596367; doi:10.1186/s12871-015-0111-z)

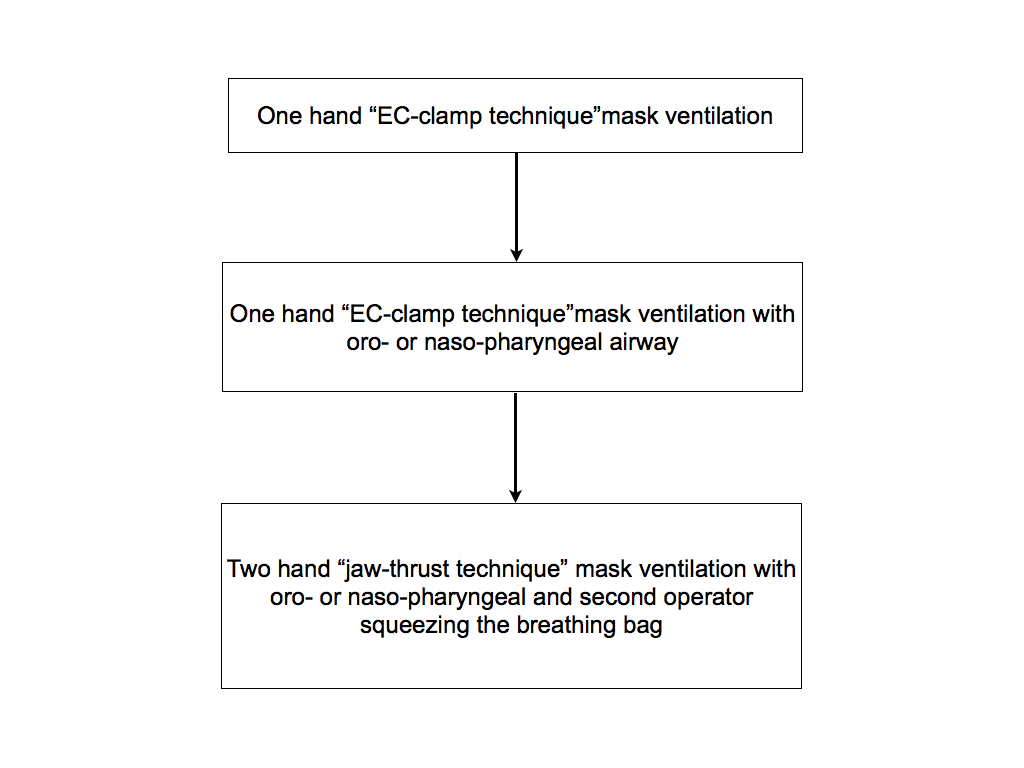

Supplement: Additional file 1: — Suggested algorithm for dealing with increasing levels of difficulty with facemask ventilation. (TIFF 3 mb) [file 12871_2015_111_MOESM1_ESM.tiff]
